# Three-stage anaerobic co-digestion of food waste and horse manure

**DOI:** 10.1038/s41598-017-01408-w

**Published:** 2017-04-28

**Authors:** Jingxin Zhang, Kai-Chee Loh, Jonathan Lee, Chi-Hwa Wang, Yanjun Dai, Yen Wah Tong

**Affiliations:** 1 NUS Environmental Research Institute, National University of Singapore, Singapore 138602, Singapore; 2 Department of Chemical & Biomolecular Engineering, National University of Singapore, Singapore 117576, Singapore; 3 Department of Civil and Environmental Engineering, National University of Singapore, Singapore 117576, Singapore; 4 School of Mechanical Engineering, Shanghai Jiao Tong University, Shanghai, PR China

## Abstract

A novel compact three-stage anaerobic digester (HM3) was developed to combine the advantages of high solids anaerobic digestion (AD) and wet AD for co-digestion of food waste and horse manure. By having three separate chambers in the three-stage anaerobic digester, three different functional zones were created for high-solids hydrolysis, acidogenesis and wet methanogenesis. The results showed that the functionalized partitioning in HM3 significantly accelerated the solubilization of solid organic matters and the formation of volatile fatty acids, resulting in an increase of 11~23% in methane yield. VS reduction in the HM3 presents the highest rate of 71% compared to the controls. Pyrosequencing analysis indicated that different microbial communities in terms of hydrolyzing bacteria, acidogenic bacteria and methanogenic archaea were selectively enriched in the three separate chambers of the HM3. Moreover, the abundance of the methanogenic archaea was increased by 0.8~1.28 times compared to controls.

## Introduction

Anaerobic digestion (AD) is an environmentally green and effective method for organic waste treatment and renewable energy recovery^1–3^. The substrates for AD are widespread, including municipal, agricultural, horticultural and industrial wastes^4, 5^. Food waste (FW) is an attractive feedstock for AD because of its high methane production potential^6^. However, AD of FW often encounters some drawbacks e.g. a suboptimal carbon to nitrogen (C/N) ratio, lack of certain nutrients and a low pH^1^. To overcome the deficiencies of mono-digestion, anaerobic co-digestion - the simultaneous AD of FW with other organic wastes, was developed to improve the operational stability and economic viability of AD plants^7–9^. A common example is to co-digest FW with animal manure since co-digestion not only provides good buffering capacity to the AD systems but the nutrient profile is also favorably altered^1, 10^.

Horse manure (HM), a mixture of faeces and bedding materials, is the major source of animal manure in Singapore. It is estimated that around 30–40 tons HM are produced from Singapore turf club every day. However, the digestibility and biochemical methane potential of HM are the lowest in most cases compared to other livestock manures as HM contains bedding materials (straw, woodchips, etc.) which have a high content of refractory organic compositions such as lignin and cellulose^11–13^. Lignin is one of the key rate-controlling factors limiting AD of lignocellulosic biomass as lignin encloses the cellulose and hence prevents the accessibility of cellulose^14^. As a result, the efficiency of co-digestion of FW and HM is limited when the content of lignin is high. To improve the biochemical methane potential of lignocellulosic biomass, several physico-chemical pretreatment methods have been applied to destroy the structure of lignin and increase the digestibility of lignocellulose before AD consequently incurring higher operating costs and energy^15, 16^. In contrast, a separate biological solubilization process has also proposed to be a cost-effective method to treat organic waste, whereby the organic particles are microbiologically hydrolyzed and the size of those particles reduced prior to AD^17^. Currently, this biological solubilization process is conducted as a pre-treatment step. In this research, we anticipated that the overall performance of AD could be improved if the hydrolyzing and fermenting process of HM and FW were conducted before methanogenesis within the same anaerobic digester. On the other hand, the transfer and digestion of large fibrous particles in manures are liable to cause technological issues like choking of pumps and pipes^18, 19^. For this reason, only liquid manures are commonly used in practical AD plants while the utilization of most of solid manures or other residues with fibrous materials is limited in conventional AD digesters.

**Figure 1:**
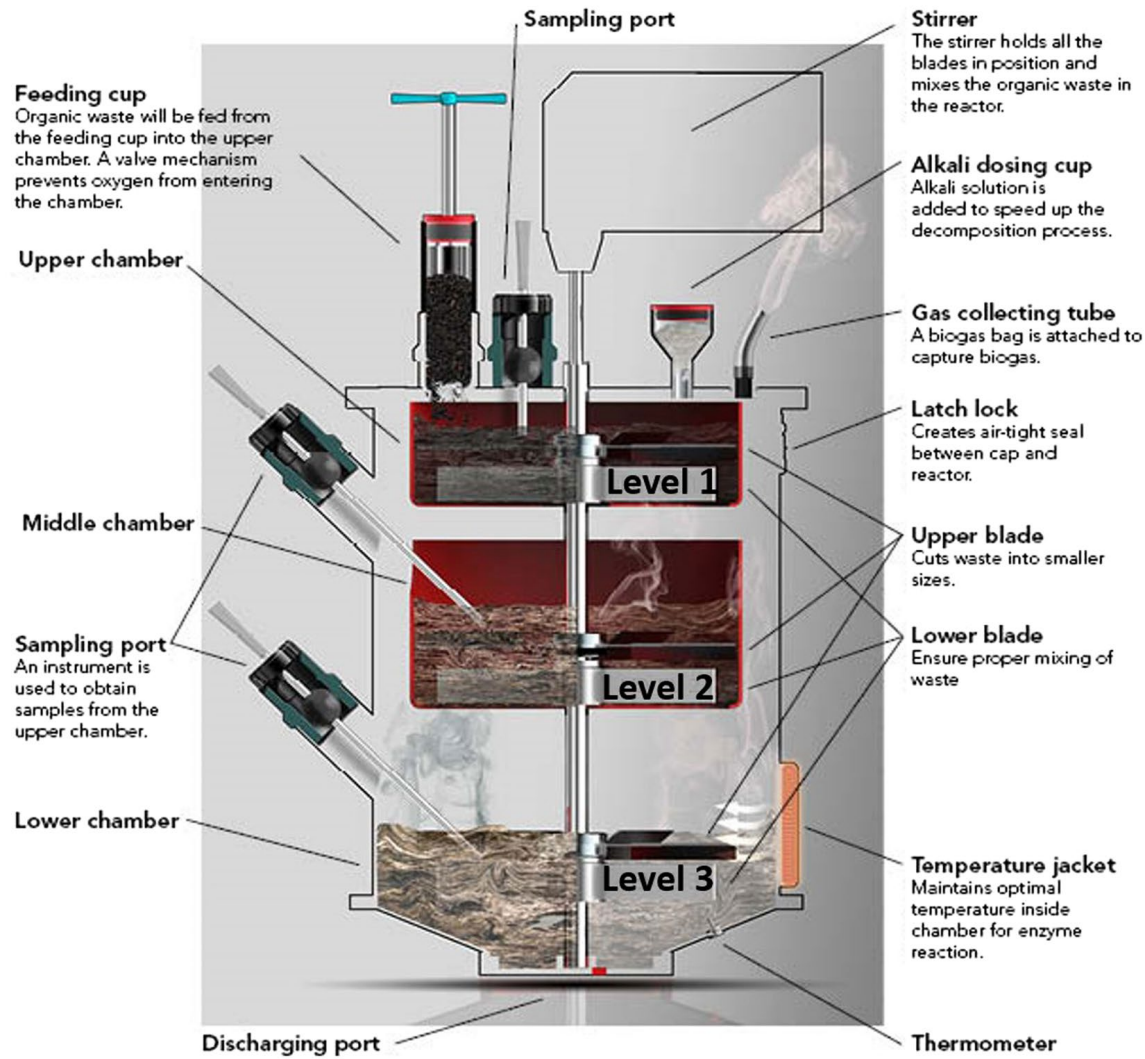
Schematic diagram of three-stage anaerobic digester (HM3).

On the basis of total solids (TS) content, AD can be categorized into wet AD (TS < 15%) and high-solids AD (15% < TS < 40%)^20^. High solids AD is preferable for reactor design as it results in a much smaller requirement for reactor volume. However, the higher moisture content of wet AD promotes the growth of methanogens and enhances mass transfer between substrate particles and microorganisms during methanogenesis. Given the issues associated with co-digestion of HM and FW, specifically the requirement to effect additional biological solubilization and the need to deal with high total solids content, we designed a three-stage anaerobic digester in which the processes of hydrolysis, acidogenesis and methanogenesis could be independently controlled yet simultaneously operated within three separate chambers (Fig. 1). In this novel design, the hydrolysis and acidogenesis chambers operated as high solids digesters, while the methanogenesis chamber operated under wet AD conditions. Hitherto, most studies only focussed on either wet AD or high-solids AD, and little research has been conducted to design and operate a compact AD reactor that combines both types in one digester. Our streamlined three-chambers design allows a smaller footprint due to its reduced volume from improved treatment capacity and efficiency. This helps to reduce land costs and capital costs. Moreover, the reactor design is compact with functional partitioning and the transfer of feedstocks between each chamber is conducted by gravity transmission, avoiding the blocking of pumps or pipes that could potentially happen with fibrous materials in the manures. In addition to energy saving from gravity transmission, a single stirrer was employed in the three chambers simultaneously through a drive rod with three blades for mixing purposes. These contribute to the transfer of large fibrous particles of feedstocks in a simple and energy saving way. The objectives of this research were to (a) evaluate the feasibility and potential of this novel anaerobic digester for anaerobic co-digestion of FW and HM and (b) to examine in-depth the interaction between bacterial and archaeal communities in our overall endeavor to enhance the AD of organic solid wastes.

## Results and Discussion

### Treatment performance of high-solids hydrolysis and acidogenesis

Hydrolysis and acidogenesis convert complex organics into volatile fatty acids (VFAs), which play an important role in the whole AD process^21^. Table 1 shows the contents of various soluble products after hydrolysis and acidogenesis. The hydrolytic efficiency can be evaluated by the degree of solubilization, which is expressed as the quotient of soluble COD versus the total COD of a solid sample^22^. The degree of acidification is defined as the ratio of COD-equivalent TVFA to the soluble COD^23^.

**Table 1:** Characteristics of feedstocks after hydrolysis and acidogenesis.

Parameter	SCOD (g COD/g VS)	TCOD (g COD/g VS)	TVFA (g COD/g VS)	Ammonia (mg/g VS)	^1^Degree of solubilization (%)	^2^Degree of acidification (%)
Fresh mixture of FW and HM	0.25 ± 0.04	1.31 ± 0.07	0.11 ± 0.01	0.58 ± 0.04	19%	44%
FW and HM after hydrolysis	0.44 ± 0.03	1.31 ± 0.07	0.22 ± 0.03	1.31 ± 0.06	34%	50%
FW and HM after hydrolysis and acidogenesis	0.59 ± 0.05	1.31 ± 0.07	0.37 ± 0.02	9.93 ± 0.1	45%	63%

Data are the averages of the values obtained. Error bars represent standard deviations of statistical analysis. Degree of solubilization is expressed as the quotient between Soluble COD (SCOD) and Total COD (TCOD)^22^. Degree of acidification is defined as the ratio of COD-equivalent TVFA to SCOD^23^.

As seen from Table 1, the degree of solubilization of FW and HM increased from 19% to 34% after 2 days of hydrolysis. Also, an increase in the degree of acidification from 44% to 50% was achieved during hydrolysis. During the acidogenesis stage, the degree of acidification increased sharply to 62.8%, increasing the production of VFAs that helps to improve substrate suitability for the subsequent methanogenic step^24, 25^. In addition, a hydrolysis yield of 45% was also achieved in acidogenesis. The above results indicate that the increased acidification yield can mainly be ascribed to the acidogenic stage while high-solids hydrolytic stage accounted for much of the improved hydrolysis yield.

As an essential nutrient for the growth of microorganisms and a buffering agent (C_x_H_y_COOH + NH_3_ × H_2_O → C_x_H_y_COO^−^ + NH_4_^+^  + H_2_O), ammonia plays an important role in the performance and stability of AD. After two days of hydrolysis, the concentration of ammonia increased from 0.58 ± 0.04 to 1.31 ± 0.06 mg/g VS. In comparison, the total amount of ammonia released in the acidogenic stage was 9.93 ± 0.1 mg/g VS, 11.8 times higher than that in the hydrolytic stage. The higher concentration of ammonia can be due to the adequate anaerobic hydrolysis and acidogenesis of the N-rich organic substrates like proteins into amino acids and then to ammonia^26^.

### Baseline comparison of HM1, HM2 and HM3

**Figure 2:**
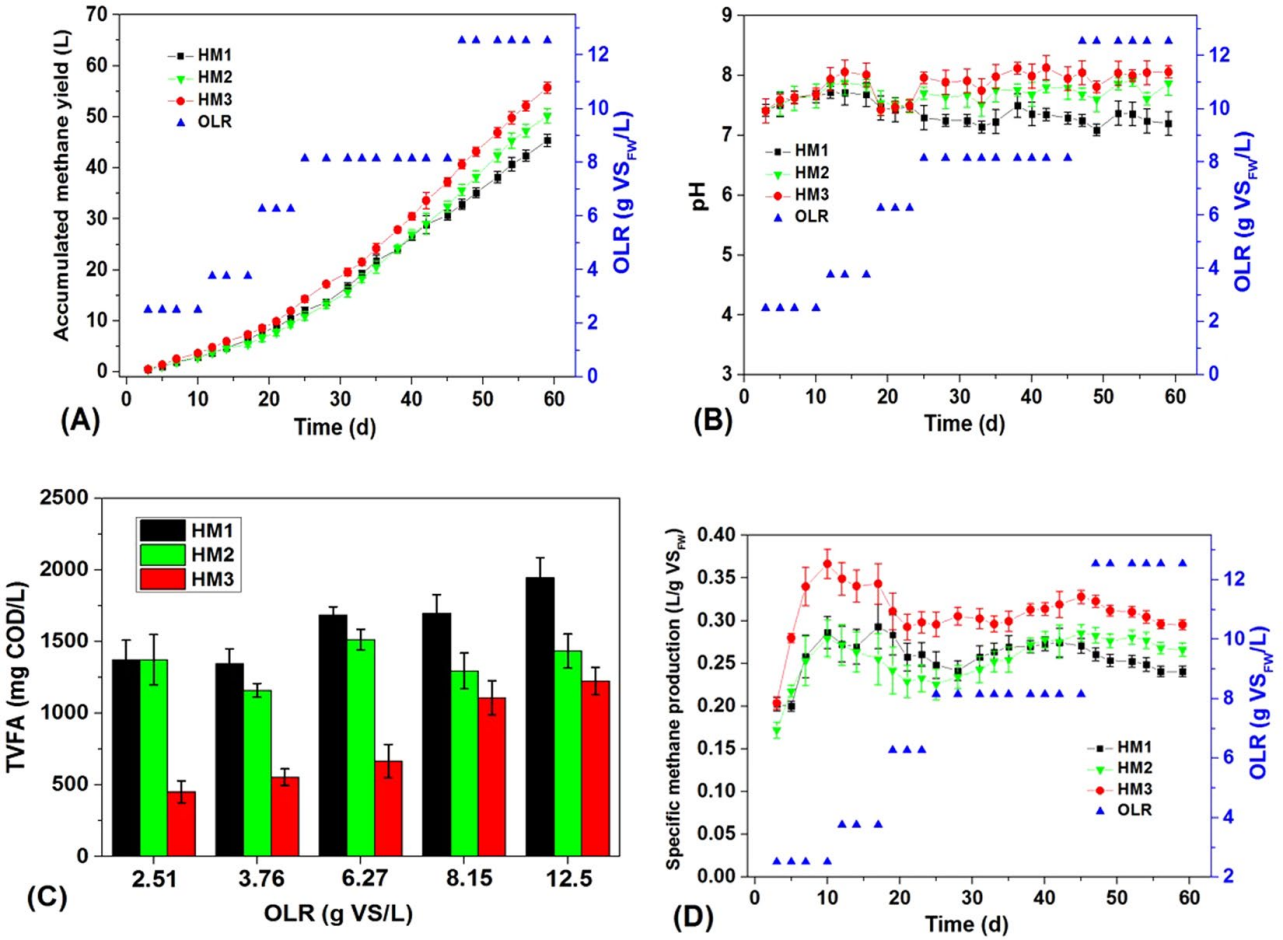
Comparison of (**A**) accumulated methane yield, (**B**) pH, (**C**) TVFA concentration and (**D**) Specific methane production among the three ADs (HM1, HM2 and HM3).

To evaluate the performance of three-stage anaerobic co-digestion of FW and HM, HM3 and two control reactors (HM1 and HM2) were operated in parallel. As shown in Fig. 2A, the accumulated methane yield increased at increasing OLR from 2.5 to 12.5 g VS/L. During the initial 20 days, the methane yields in the three digesters were hardly significant as the low OLR of 2.5 to 3.76 g VS/L. After 60 days of operation, the accumulated methane yield of HM3 increased to 55.7 L at an OLR of 12.5 g VS/L, 22.7% higher than the 45.4 L in HM1. The enhanced methane production potential is seems to be attributed to the increased degree of solubilization and acidification. In comparison, HM2 has 11.8% higher methane yield than that of HM1 due to the hydrolyzing process of FW and HM. Compared to HM2, the accumulated methane yield was 10.9% higher in HM3, which might be attributed to the positive effect of acidogenesis that increased the VFAs production and further enhanced the solubilization of FW and HM (Table 1). These results indicated that the hydrolysis and acidogenesis in HM3 contributed to 11.8% and 10.9% of the improved methane yield, respectively. Moreover, HM3 still presented the highest value in daily specific methane production (SMP) among the three AD reactors during the whole AD process, which reached approximately 0.3 L/gVS after 60 days of operation, indicating that hydrolysis and acidogenesis would enhance the subsequent methanogenic activity and improve methane yield.

From Fig. 2B, the pH value of HM1, HM2 and HM3 kept between 7.09 and 8.05 during the whole experiments. Compared with HM1, both HM2 and HM3 had a slightly higher pH value. However, the pH value in these ADs was still within the proper range for methanogenesis even at a high OLR^27^. This result was in agreement with Zhang *et al*.^10^ who found that the AD system stability was improved when using co-digestion of FW and cattle manure because of the high buffering capability of cattle manure while AD fed only with FW soured and failed. Figure 2C shows the variations of VFAs concentration in the three digesters. During 60 days of operation, the VFA concentration in HM3 started at 453 mg COD/L initially and increased to 1226 mg COD/L. However, it was still much lower than the 1434 mg COD/L in HM2 and 1946 mg COD/L in HM1. These results further confirmed that the improved efficiency of methane production in HM3 might be mainly attributed to the enhanced hydrolysis of organic particles in FW and HM and VFAs production but not the pH decline.

### Final AD performance and quantification of microorganisms

After 60 days of operation, the VS reduction in the HM3 was 71%, which was much higher than the 62% and 57% in the HM1 and HM2, respectively (Table 2). As shown in Table S1, the average contents of recalcitrant organics like cellulose, hemicellulose and lignin in HM were 33.7%, 22.1% and 16.9%, respectively, which were much higher than the 8.5%, 2.8% and 6.8% in FW. Comparing to the easily digestibility of FW, high contents of cellulose, hemicellulose and lignin in lignocellulosic biomass like HM are usually with low digestibility^28^, passing undigested through AD process. This might be a possible reason for the accumulation of VS in the residues of AD. However, HM3 still present the highest VS reduction comparing to HM1 and HM2. It has been reported that phase separation of AD can result in a 1.9~6% increase in VS reduction for sludge digestion over conventional one-stage AD^29^, which might be a possible reason for the enhanced VS reduction in HM3 due the three separate chambers of high-solids hydrolysis, acidogenesis and methanogenesis.

**Table 2:** Variations of VS reduction, pH, 16 S rRNA gene copies of bacteria and archaea, methane yields and methane content in three ADs after 60 days of operation.

Digester	OLR (g VS_FW_/L)	pH	VS Reduction (%)	16 S rRNA gene copies/g TS		Biogas	
				Bacteria	Archaea	CH_4_ content (%)	Yield L CH_4_/g VS/L
HM1	12.5	7.3 ± 0.1	57 ± 2	1.5 ± 0.1 × 10^9^	5.0 ± 0.2 × 10^7^	55 ± 3.7	0.3 ± 0.01
HM2	12.5	7.7 ± 0.2	62 ± 3	1.8 ± 0.2 × 10^9^	6.4 ± 0.3 × 10^7^	60 ± 5.9	0.34 ± 0.09
HM3	12.5	7.9 ± 0.1	71 ± 3	2.0 ± 0.3 × 10^9^	1.1 ± 0.2 × 10^8^	63.9 ± 7.5	0.37 ± 0.01

Values are expressed as mean value ± standard deviations.

At an OLR of 12.5 g VS/L, HM3 showed the highest average methane yield of 0.37 L CH_4_/g VS comparing to HM2 and HM1, with CH_4_ content of 57.6~70.1%. Theoretically anaerobic methane production can be a maximum of 350 ml/g COD_removed_^30^. From Table 1, 1 g VS_FW+HM_ corresponds to 1.31 g COD. If the conversion rate of COD to CH_4_ is 100%, the theoretical methane production per g VS will be 1.31 × 350 ml = 458.5 ml i.e. 0.458 L CH_4_/g VS_FW+HM_. After 60 days of operation, the methane yield of HM3 is much close to the theoretical methane yield. The losses of methane yield might be mainly attributed to the incomplete degradation of FW and HM in the sludge phase and the residual soluble organics like VFAs in the liquid phase (Fig. 2C and Table 2). However, the methane yield in HM3 is much higher than that obtained from HM1 and HM2.

Real-time PCR analysis showed that the number of copies of the archaeal 16 S rRNA in HM3 was 1.1 × 10^8^ copies/g TS, a 0.8-fold and 1.28-fold higher archaeal abundance than that of HM2 and HM1, respectively. Generally, archaea account for most of the methanogens in AD. The enrichment of archaea in HM3 was in agreement with its high methane yield (Fig. 2). Moreover, HM3 (2.0 × 10^9^ copies/g TS) also presented a slightly higher bacterial abundance than those of HM2 (1.8 × 10^9^ copies/g TS) and HM1 (1.5 × 10^9^ copies/g TS).

### Bacterial community

After pyrosequencing, a total of 21524 (HM1), 24306 (HM2), 20013 (HM3) and 38768 (seed sludge) effective reads were obtained. From Table S2, the observed operational taxonomic units (OTUs) were 1217 (HM1), 1279 (HM2), 1520 (HM3) and 7054 (seed sludge) respectively, suggesting that reactor HM3 had the most species richness among the three digesters. Alpha diversity analysis in terms of Chao1, Shannon and ACE indices showed that the biodiversity in HM3 was much higher than that of HM2 and HM3 with a coverage of 0.96~0.97. It has been reported that high biodiversity can enhance the ecological stability, which is the maintenance of performance of AD under a change of environmental conditions^31^.

**Figure 3:**
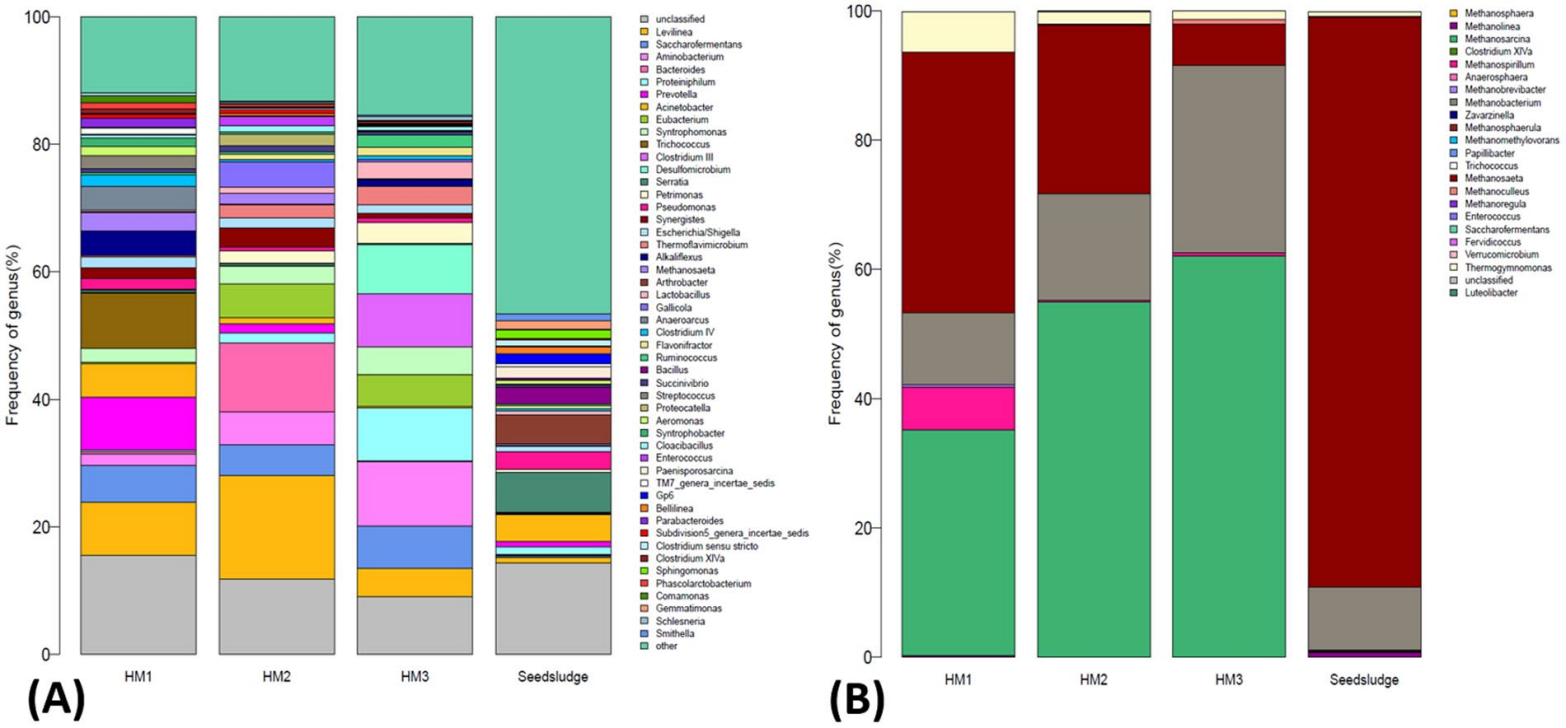
(**A**) Taxonomic classification of the dominant phylogenetic groups from HM1, HM2, HM3 and Seed sludge at the genus levels, (**B**) Taxonomic classification of the dominant archaeal groups from HM1, HM2, HM3 and Seed sludge at the genus levels.

As shown in Fig. 3A, the relative abundances of species were identified at the genus level. More than 31 different dominant species were identified in the three digesters. Nine dominant species i.e. *Aminobacterium* (10.1%), *Clostridium* (8.3%), *Proteiniphilum* (8.4%), *Saccharofermentans* (6.7%), *Eubacterium* (5%), *Syntrophomonas* (4.3%), *Petrimonas* (3.3%), *Thermoflavimicrobium* (2.9%) and *Flavonifractor* (1.4%) in HM3 were the main difference in bacterial communities among the three digesters. *Aminobacterium* belongs to amino acid fermenting bacteria which are capable of transforming amino acids to ammonia. It was usually co-localised with hydrogenotrophic methanogens to oxidize alanine, glutamate and leucine^32^. Since ammonia can buffer VFAs and prevent pH decreasing according to the following reaction: C_x_H_y_COOH + NH_3_ × H_2_O → C_x_H_y_COO^−^ + NH_4_^+^  + H_2_O^33^, the *Aminobacterium* might help to enhance the buffering ability of HM3 to tolerate the high OLR. *Proteiniphilum* and *Petrimonas* are known to play an important role in converting various organic compounds to acetic acid and CO_2_^34, 35^. Their presence might help to optimize VFAs composition and accelerate methane production by driving the formation of acetic acid and CO_2_. *Syntrophomonas*, an obligate anaerobic and syntrophic bacteria, has the ability to oxidize saturated fatty acids, which is expected to enhance VFAs consumption. *Clostridium, Saccharofermentans*, *Eubacterium*, *Thermoflavimicrobium* and *Flavonifractor* are all strict anaerobic bacteria, and most of them are acidogenic bacteria capable of fermenting sugars, lipids and proteins to VFAs.

### Archaeal communities

Compared with the bacterial community (Fig. 3A), overall archaeal biodiversity is low. From Fig. 3B, a total of 12 genus were identified in the three digesters, in which only four archaea i.e. *Methanosarcina*, *Methanobacterium*, *Methanosaeta* and *Thermogymnomonas* were dominant, accounting for 92.6~99.6% of total archaea. The relative abundance of *Methanosarcina* in HM3 was 62.1% while it was 54.9% in HM2 and 34.9% in HM1. *Methanosarcina* is a multi-functional methanogen which can generate CH_4_ through three different metabolic pathways using H_2_/CO_2_, acetate and methylated one-carbon compounds as substrates^36^. The enrichment of *Methanosarcina* may help to accelerate the consumption of various intermediates and improve methane production. The relative abundance of hydrogen-utilizing methanogen *Methanobacterium* in HM3 (28.9%) was 0.76-fold and 1.61-fold higher than that in HM2 and HM1 respectively. It is known that hydrogen-utilizing methanogens play a key role in producing methane (approximately 30% of CH4 is formed from H_2_/CO_2_) and in maintaining a low partial pressure of H_2_ in the AD process, which is necessary for the growth of syntrophic bacteria^37, 38^. The enrichment of syntrophic bacteria including *Proteiniphilum*, *Petrimonas* and *Syntrophomonas* in HM3 was in tangent with the enrichment of *Methanobacterium*. An acetate-utilizing methanogen (*Methanosaeta*) was in relatively high abundance in seed sludge and HM1. *Thermogymnomonas* was also dominant in HM1 while its relative abundance in HM2 and HM3 was low. Even though *Thermogymnomonas* belongs to archaea in the order of *Thermoplasmatales* ^39^, it is not a methanogen and cannot produce CH_4_, which might be a possible reason for the lower methane yield in HM1.

### Functional partitioning in three-stage AD

AD requires the concerted action of groups of hydrolyzing bacteria, acidogenic bacteria and methanogens, each performing their particular role in the overall degradation process. The functional partitioning in this three-stage AD system is expected to create three different favorable conditions for selectively enriching each trophic group of microbes for high solids hydrolysis, acidogenesis and wet methanogenesis.

**Figure 4:**
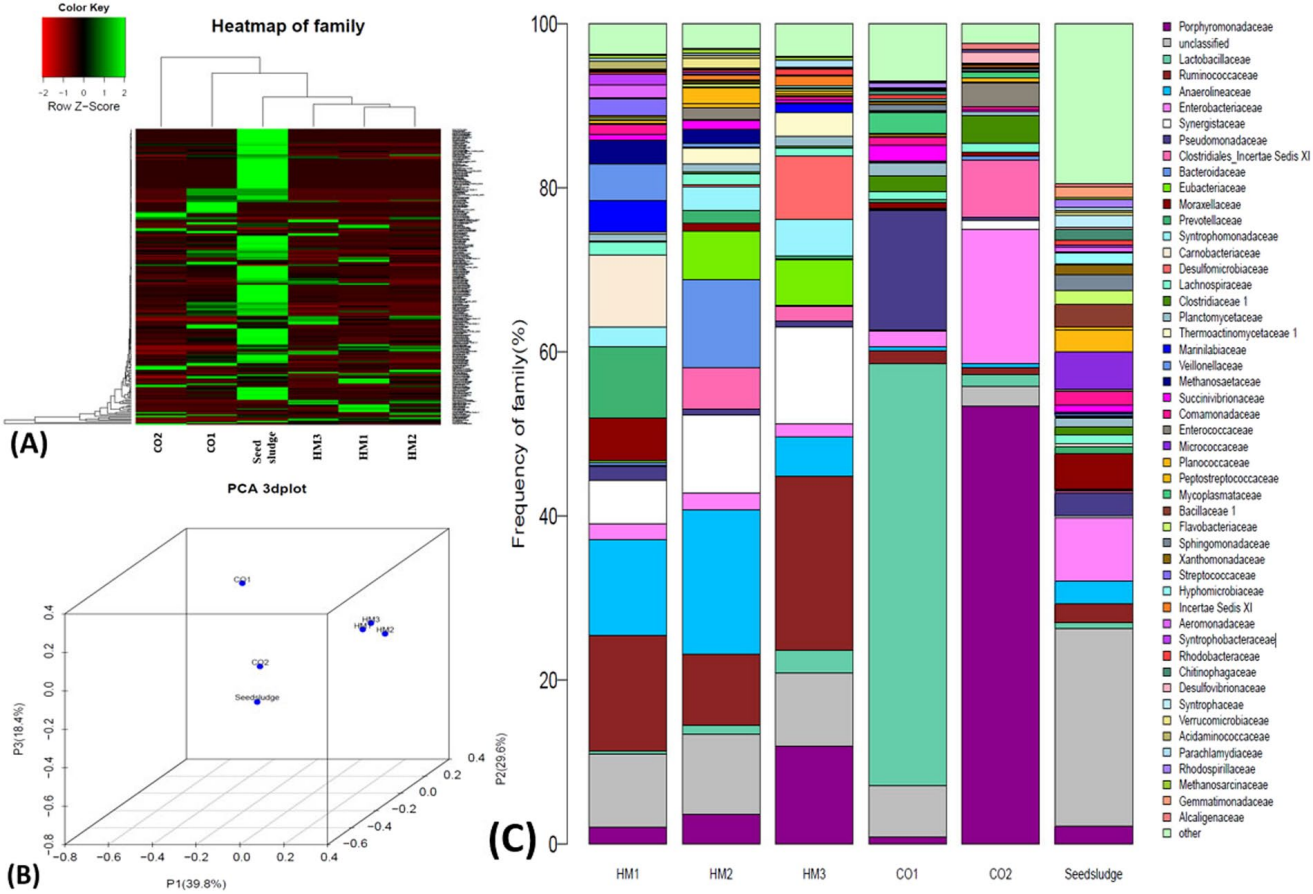
(**A**) Hierarchical cluster analysis of bacterial communities in the sludge samples of high-solids hydrolytic stage (CO1) of HM3, high-solids acidogenic stage (CO2) of HM3, HM1, HM2, HM3 and seed sludge. The OTUs were instructed at family level. Sample communities were clustered according to the method of complete linkage. The colour intensity of scale indicates relative abundance of each OTU read. Relative abundance was defined as the numbers of sequences affiliated with that OTU divided by the total number of sequences per sample. (**B**) 3D-plot of principal component analysis (PCA) analysis of bacterial communities. (**C**) Taxonomic classification of the dominant phylogenetic groups from high-solids hydrolytic stage (CO1) of HM3, high-solids acidogenic stage (CO2) of HM3, HM1, HM2, HM3 and seed sludge at the family level.

As shown in Fig. 4, the bacterial community structures established in high-solids hydrolysis, acidogenesis, HM1, HM2 and HM3 were compared. Hierarchical cluster analysis showed that the cluster of bacterial population in high-solids hydrolytic stage (CO1), acidogenic stage (CO2) and HM3 were separated, suggesting that the dominant bacterial populations in each stage were significantly different (Fig. 4A). PCA analysis was used to compare difference metrics among the multiple groups based on Unifrac. In Fig. 4B, HM1, HM2 and HM3 were clustered together, away from CO1 and CO2, indicating that the species composition between CO1, CO2 and the group of HM1, HM2 and HM3 have low sequence similarity. To identify the different composition, an analysis of the species was conducted at the family level.

As seen in Fig. 4C, *Lactobacillaceae* (51.5%) and *Pseudomonadaceae* (14.6%) were two predominant hydrolyzing bacteria in CO1. The members of *Lactobacillaceae* are facultative anaerobic bacteria, with an ability to ferment carbohydrates to lactate and other by-products e.g. acetate, ethanol, formate and succinate. *Pseudomonadaceae* are heterotrophic bacteria, with the ability to actively oxidise carbohydrates and breakdown aromatic rings, and are able to convert sugars into their biomic acids^40^. It is likely that the enrichment of these two families contributed to the decomposition and solubilization of FW and HM in the high-solids hydrolytic stage.

**Figure 5:**
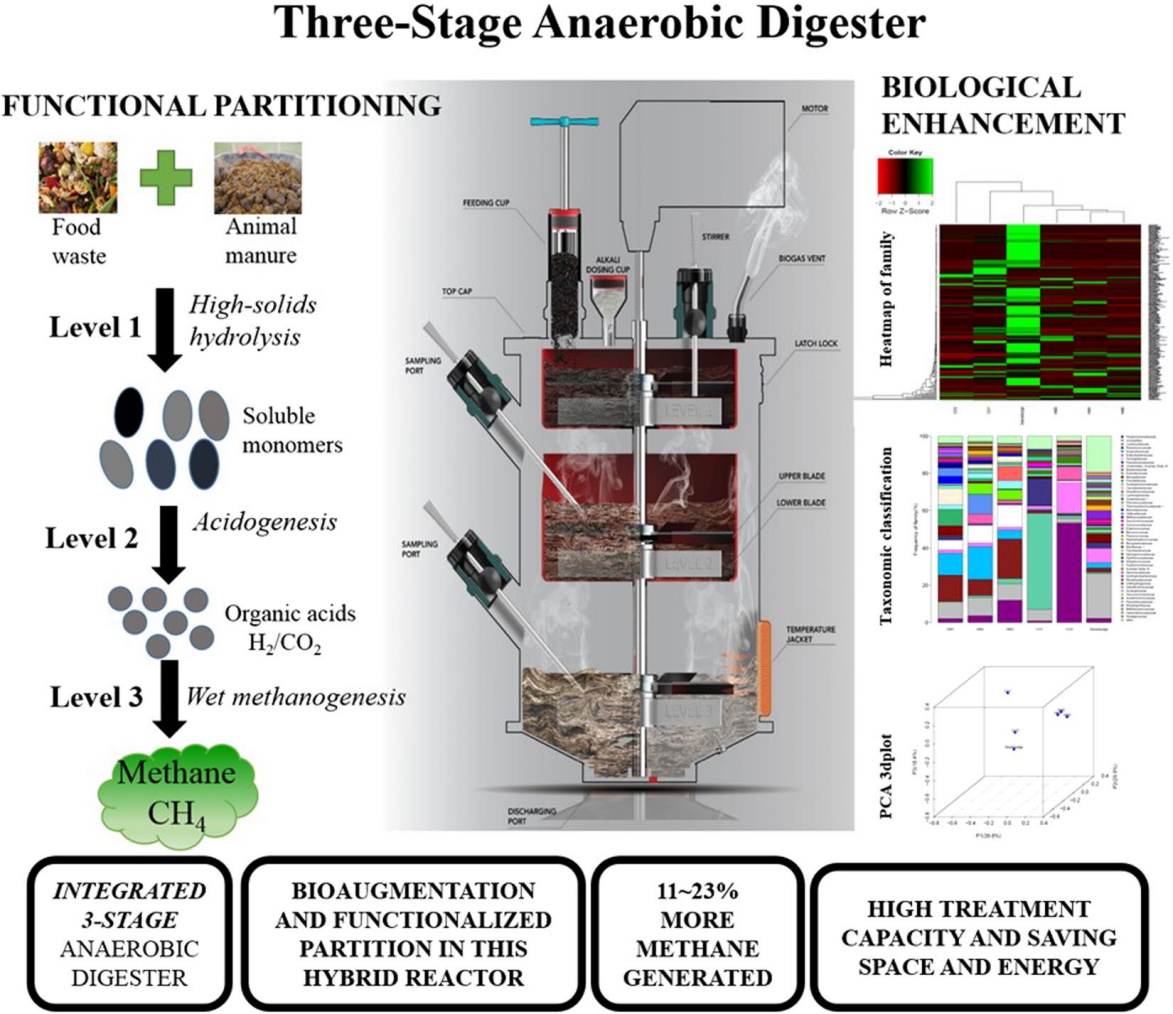
Conceptual graph of three-stage anaerobic digester (HM3).

Compared to CO1, the dominant species in CO2 were different. The results showed that *Porphyromonadaceae* (53.4%) and *Enterobacteriaceae* (16.4%) were the dominant bacteria, accounting for almost 70% of the total bacteria. *Porphyromonadaceae*, commonly found in AD, are capable of degrading complex carbohydrates and proteinaceous compounds and catalyzing the production of VFAs^41^. This is supported by the fact that its enrichment was in sync with the increased NH_4_^+^ and VFAs concentration during acidogenic stage of CO2. *Enterobacteriaceae* are facultative anaerobes, fermenting sugar to lactic acid and various other end products. However, no methanogenic species were identified in CO1 and CO2. In contrast, both bacterial and methanogenic populations were enriched in HM3 (as discussed in section3.4.1). On basis of the above results, the conceptual graph was summarized in Fig. 5.

### Preliminary test of a small-scale HM3

**Figure 6:**
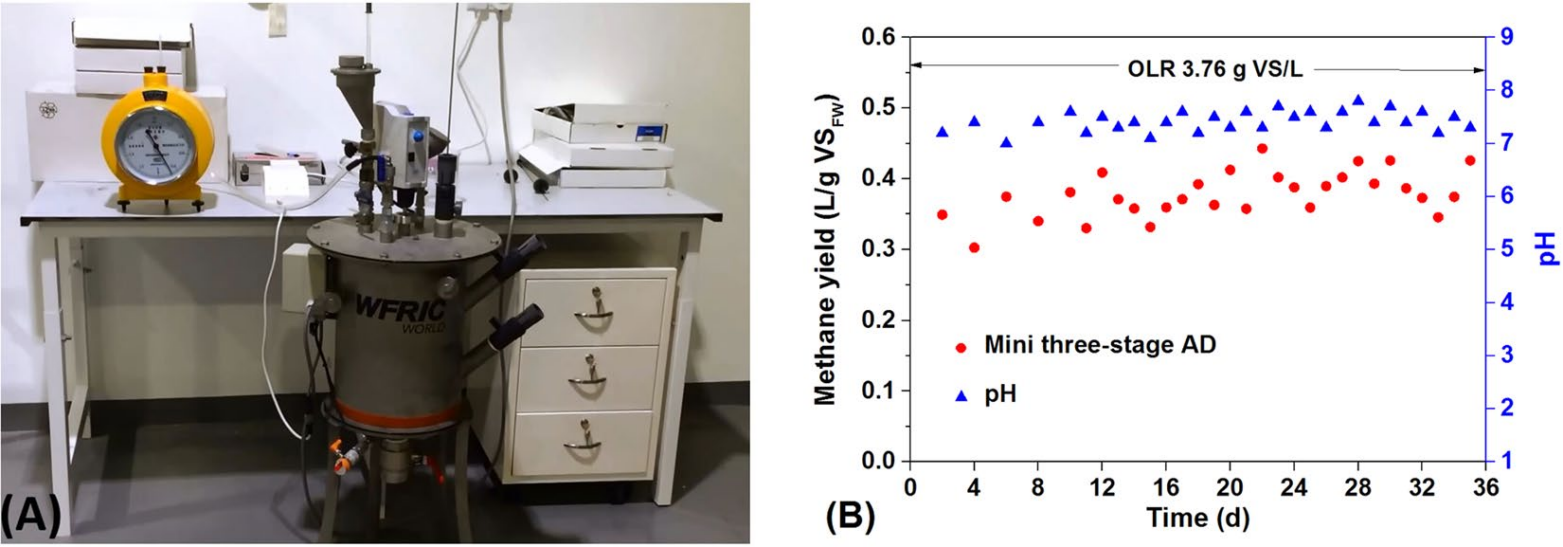
(**A**) A 20 L mini three-stage anaerobic digester system; (**B**) Methane yield and pH.

The above baseline studies confirmed that the treatment capacity and methane production potential of HM3 is much higher than that of traditional one-stage and two-stage ADs. After testing bench-scale anaerobic digesters, a 20 L small-scale HM3 was fabricated and tested for the co-digestion of FW and HM. From Fig. 6, the operation of this small-scale HM3 was rather stable at an OLR of 3.76 g VS_FW+HM_/L. After 35 days’ operation, the methane yield reached 0.38 ± 0.03 L CH_4_/g VS_FW+HM_/d, and the pH was maintained at 7.4 ± 0.2. The VS reduction of this small-scale HM3 reached 73%. Some studies reported that the average methane yield on anaerobic co-digestion of FW and animal manure e.g. chicken manure or cattle manure ranged from 0.32 to 0.67 L CH_4_/g VS/d^1, 10, 11, 42^. However, most of these studies are wet AD, which suffers from the need for larger reactor volume and high consumption of water. In comparison, HM3 is a highly compact AD with three separate chambers, reducing the reactor volume requirement. The required seed sludge/water in the high-solids hydrolytic and acidogenic stage can be obtained from the waste sludge recycle from methanogenic stage. Since methanogenesis can produce alkali through consuming VFAs, it might also be favorable for buffering acids produced from high-solids hydrolysis and acidogenesis to maintain a favorable pH. However, compared to the ideal methane yield^1, 10, 11, 42^, there is still room for the optimization of reactor structure, operational parameters and inocula selection in the furture work.

### Conclusions

A compact three-stage anaerobic digester has been fabricated and tested for enhancing anaerobic co-digestion of FW and HM and methane production. Results of baseline studies show that the methane yield was increased by 11.2~22.7% in HM3 compared to the controls. The separate three chambers in the three-stage AD enhanced the hydrolytic and acidogenic efficacy of solid organic matters, accelerating the subsequent methanogenesis for methane production. Different microbial communities were enriched selectively in each stage of HM3 in a form of functional partitioning. This compact anaerobic digester can be considered as coupling high-solids AD to wet AD, which is favorable for improving OLR, methane yield and reducing digester volume.

## Materials and Methods

### Experimental set-up and reactor specifications

In this study, the development process of the three-stage anaerobic digester (HM3) includes reactor design, baseline study, and device fabrication and test. Figure 1 shows the schematic diagram of HM3 for this research. Chambers level 1, level 2 and level 3 correspond to high-solids hydrolysis stage, acidification stage and wet methane-production stage, respectively. FW and HM (wet mass ratio 1:1) was first fed into chamber level 1 for hydrolysis. After that, the hydrolyzed FW and HM was transferred to chamber level 2 for acidogenesis. Finally, the hydrolyzed and acidified FW and HM was dropped to chamber level 3 for methanogenesis. The optimized pH in each chamber was adjusted and controlled individually.

Before fabricating a prototype-scale steel HM3 for proof-of-concept, baseline studies involving 1 L AD reactors were carried out to examine the individual stages of hydrolysis, acidogenesis and methanogenesis. Essentially, three 1 L glass reactors (effective working volume of 0.8 L) was set up in parallel, to simulate a one-stage AD (HM1), a two-stage AD (HM2) and the HM3. FW and HM was adjusted to 20% TS through the addition of seeding sludge and then stored in the −20 °C freezer as feedstock for HM1. During the hydrolysis stage, HM1 was operated at 35 °C with a stirrer speed of 150 rpm. After 2 days of operation, the hydrolyzed FW and HM was stored in the freezer at −20 °C to be used as feedstock for HM2. For HM2 operation, this hydrolyzed FW and HM was mixed with seeding sludge to a TS of 10%. pH was controlled at 6.5 ± 0.2, to provide the optimal conditions for acidogenesis. After 2 days of operation, the acidified FW and HM was stored in the freezer at −20 °C to be used as feedstock for HM3. After adding the seed sludge, the three reactors (HM1, HM2 and HM3) were operated in a semi-continuous mode with gradual increase in organic loading rates (OLR) of 2.51, 3.76, 6.27, 8.15 and 12.54 g VS/L. All the experiments were conducted in triplicates at the same experimental conditions.

### Device fabrication

After the baseline studies, a 20 L steel HM3 (diameter 250 mm * height 400 mm) was fabricated and operated as a proof of concept. The internal structure of this HM3 system is shown in Fig. 1. This digester were operated in a semi-continuous mode at a fixed temperature of 35 °C. Fresh FW and HM were added into the digester from the top chamber once a day. The baffle at the bottom of each chamber can be opened by a connecting rod from the outside of the digester. In this way, FW and HM were gravity transferred from one chamber to another. The digestate was discharged from the bottom of HM3 every day. The mixing of each chamber can be conducted simultaneously by a central stirrer. Details of operating conditions of each step was described above. The OLR was maintained at 3.76 g VS_FW_/L.

### Inoculum and substrates

The seed sludge was collected from a large-scale anaerobic digester at Ulu Pandan Water Reclamation Plant in Singapore. The ratio of volatile suspended sludge (VS) to total suspended sludge (TS) was 0.75 with initial TS of 13.2 g/L.

FW was obtained from a canteen in the National University of Singapore, and mainly consisted of rice, noodles, meat, vegetables and condiments. HM was obtained from the Singapore turf club. After removing any bones and non-biodegradable waste like plastic bags, FW and HM was homogenized in a blender and stored at −20 °C. The detailed characteristics of FW and HM are listed in Table S1.

### Analytical methods

COD and ammonia were determined using colorimeter (HACH DR900, USA) according to the manufacturer’s instructions. The pH was recorded using a pH analyzer (Agilent 3200 M, USA). TS and VS were determined based on the weighing method after being dried at 103–105 °C and burnt to ash at 550 °C. The CH_4_ production was determined using a gas chromatograph (Clarus 580 Arnel, PerkinElmer, USA) equipped with a thermal conductivity detector. VFAs in terms of acetic acid, propionic acid and butyric acid were determined by a gas chromatograph (Clarus 580GC, PerkinElmer, USA) equipped with a flame ionization detector. C and N element contents in FW and HM were determined using a vario MICRO cube (Elementar, Germany). The contents of cellulose, hemicelluloses, and lignin were analyzed using the method proposed by Jung *et al*.^43^. The abundance of bacteria and archaea were determined by real-time PCR according to the method described by Zhang *et al*.^44^.

### DNA extraction and high - through put 16 S rDNA gene pyrosequencing

The genomic DNA of the sample was extracted using an extraction kit (MO BIO Laboratories, Inc. Carlsbad, USA) according to the manufacturer’s instructions. The quality of the extracted DNA was checked by determining its absorbance at 260 nm and 280 nm.

The diversity of microbial communities was deeply investigated by IIIumina Hiseq 2000 pyrosequencing technology. A set of bacterial primers 341 F (5′–CCTACGGGNGGCWGCAG-3′) and 805 R (5′-GACTACHVGGGTATCTAATCC-3′) was used to amplify the hypervariable V3 – V4 region of bacterial 16 S rRNA gene using net PCR. The first round archaeal primers were 340 F (5′–CCCTAYGGGGYGCASCAG-3′) and 1000 R (5′-GGCCATGCACYWCYTCTC-3′). The second round archaeal primers were 349 F (5′– GYGCASCAGKCGMGAAW-3′) and 806 R (5′-GGACTACVSGGGTATCTAAT-3′). After being purified and quantified, the PCR products of V3-V4 region of 16 S rRNA gene were determined by pyrosequencing using the IIIumina Hiseq 2000 sequencer (Sangon Biotech Shanghai Co., Ltd. People’s Republic of China). To obtain the effective sequencing data, raw pyrosequencing results were processed as follows: (1) check the completeness of the barcodes and the adapter; (2) remove sequences containing ambiguities (“Ns”); (3) remove sequences shorter than 200 bps, and (4) remove low - quality sequence i.e. a sequencing quality value lower than 20. Subsequently, effective sequences were clustered into operation taxonomic unit (OTUs) by a 3% or 5% distance level. Rarefaction curves, Shannon diversity index, species richness estimator of Chao1 and Coverage index were conducted by MOTHUR to identify the species diversity for each sample. The OTUs defined by a 3% distance level were classified using the RDP-II classifier at a 50% confidence threshold^45^.

## Electronic supplementary material

Supplementary material
