# Supplementary material for: Three-stage anaerobic co-digestion of food waste and horse manure
Source: Sci Rep. 2017 Apr 28;7:1269. doi: 10.1038/s41598-017-01408-w (PMC5430929; doi:10.1038/s41598-017-01408-w)
Supplement: Supplementary file 1 — Supplementary material [file 41598_2017_1408_MOESM1_ESM.doc]

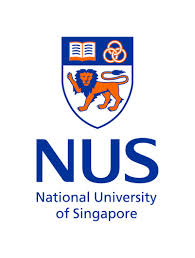


THE NATIONAL UNIVERSITY *of* SINGAPORE

Department of Chemical and Biomolecular Engineering

4 Engineering Drive 4, Singapore 117576

Three-Stage Anaerobic Co-digestion of Food Waste and Horse Manure

Jingxin Zhang^1^, Kai-Chee Loh^2^, Jonathan Lee^1, 3^, Chi-Hwa Wang^2^, Yanjun Dai^4^, Yen Wah Tong ^1, 2*^

^1^NUS Environmental Research Institute, National University of Singapore, Singapore. ^2^Department of Chemical & Biomolecular Engineering, NUS, Singapore. ^3^Department of Civil and Environmental Engineering, NUS, Singapore. ^4^School of Mechanical Engineering, Shanghai Jiao Tong University, Shanghai, PR China. Correspondence and requests for materials should be addressed to Yan Wah Tong (email: chetyw@nus.edu.sg)

Supplementary Material

| Parameters | Food waste | Horse manure |
| --- | --- | --- |
| TS (wt. %) | 33.8 ± 0.3 | 24.7 ± 0.4 |
| VS (wt. %) | 32.1 ± 0.2 | 18.1 ± 0.5 |
| VS/TS ratio | 0.95 | 0.73 |
| Lignin (%) | 6.8 ± 0.2 | 16.9 ± 0.3 |
| Cellulose (%) | 8.5 ± 0.4 | 33.7 ± 0.5 |
| Hemicelluloses | 2.8 ± 0.3 | 22.1 ± 0.5 |
|  |  |  |
| Elemental analysis (%) |  |  |
| Carbon | 49.5 ± 0.3 | 37.3 ± 0.4 |
| Hydrogen | 8.2 ± 0.2 | 5.1 ± 0.2 |
| Nitrogen | 2.3 ± 0.3 | 2.0 ± 0.3 |
| Sulfur | 0.6 ± 0.1 | 0.5± 0.1 |
| C/N ratio | 21.5 | 18.6 |

**Table S1 Characteristics of food waste and horse manure.** Values are

expressed as mean value ± standard deviations;

| Parameters | Effective reads | Observed OTUs | Chao1  Index | Shannon | ACE | Coverage |
| --- | --- | --- | --- | --- | --- | --- |
| HM1 | 21524 | 1217 | 2368 | 4.63 | 2939 | 0.97 |
| HM2 | 24306 | 1279 | 2908 | 4.63 | 4127 | 0.97 |
| HM3 | 20013 | 1520 | 3042 | 5.02 | 4262 | 0.96 |
| Seed sludge | 38768 | 7054 | 10292 | 7.20 | 10871 | 0.92 |

**Table S2. Biodiversity estimation of 16S rRNA gene libraries from the pyrosequencing**
